# High-sensitivity cardiac troponin I assays in the diagnosis of acute myocardial infarction

**DOI:** 10.1136/heartasia-2016-010867

**Published:** 2017-04-07

**Authors:** Andrew R Chapman, David E Newby, Nicholas L Mills

**Affiliations:** BHF Centre for Cardiovascular Science, University of Edinburgh, Edinburgh, UK

**Keywords:** CORONARY ARTERY DISEASE

Chest pain is one of the most common presenting symptoms in patients attending the emergency department; yet, less than one in five patients receive a final diagnosis of acute myocardial infarction.^1^ In patients without ST segment elevation, early exclusion of acute coronary syndrome has the potential to reduce healthcare expenditure in a population with significant resource implications. Previous approaches were hindered by the performance of contemporary troponin assays, which used a decision threshold for the diagnosis of myocardial infarction based on assay imprecision rather than a defined upper reference limit from a healthy population.^2^ High-sensitivity cardiac troponin assays were first introduced into clinical practice in Europe, Canada, Australia and New Zealand in 2010, and are defined by their ability to measure circulating troponin in the majority of healthy individuals, allowing accurate identification of the reference range, and demonstrating an acceptable level of imprecision at the 99th centile (≤10% coefficient of variation): the recommended decision threshold for acute myocardial infarction in clinical practice.^3^ Importantly, international guidelines now recommend these assays may be used to rule out the diagnosis of myocardial infarction at an earlier stage which may prevent unnecessary hospital admission.^3^ ^4^ However, as there are a number of different high-sensitivity cardiac troponin assays from different manufacturers, each with their own strengths and limitations, it is essential clinicians are aware of their unique characteristics to allow optimal and safe use in clinical practice.

In their expert consensus document, Tan *et al*^5^ provide a systematic approach to the optimal use of high-sensitivity cardiac troponin I assays for the Asia-Pacific region, including a hybrid diagnostic algorithm for both rule-in and rule-out of myocardial infarction. In line with other international guidelines, they recommend cardiac troponin as the biomarker of choice in patients with suspected acute coronary syndrome, with concentrations measured using a high-sensitivity assay and reported in ng/L to encourage consistency and minimise interpretation error. To confirm or exclude a diagnosis of myocardial infarction, Tan *et al* recommend serial cardiac troponin testing in all patients, although alternative approaches do exist.

The latest European Society of Cardiology guidelines allow rule-out of acute coronary syndrome in patients with at least 6 hours of symptoms who are <99th centile on presentation to hospital.^4^ However, there are concerns that the 99th centile is not the optimal threshold to safely rule out myocardial infarction in the absence of serial testing, with recent studies showing this approach missed a number of index diagnoses.^6^

There are now a number of validated studies demonstrating a single high-sensitivity cardiac troponin I or T test on presentation may reliably exclude the diagnosis of myocardial infarction with high negative predictive value. Such approaches use a cardiac troponin decision threshold far below the 99th centile, often at or close to the limit of detection, exploiting the robust performance of high-sensitivity assays. For example, a high-sensitivity cardiac troponin I concentration <5 ng/L gave a negative predictive value of 99.6% in 6304 consecutive patients presenting with suspected acute coronary syndrome.^1^ Additional strategies using lower concentrations of cardiac troponin I such as the limit of detection (1.9 ng/L) or higher have been proposed.^7^ These strategies reduce the number of patients eligible for rule-out and may reduce the absolute number of missed cases. Whether single rule-out strategies using low concentrations of cardiac troponin are safe and effective in clinical practice require further prospective evaluation in randomised controlled trials. Until then, serial testing strategies will remain a critical component of the evaluation of patients with suspected acute coronary syndrome.

Tan *et al* recommend serial testing on presentation and at 3 hours in line with other international guidelines.^3^ ^4^ In this setting, the authors suggest a change of >50% at 3 hours is indicative of a patient at high risk of acute myocardial infarction. This is broadly in line with prior recommendations from the European Society of Cardiology Working Group on Acute Cardiac Care, and is thought to provide sufficient clarity from both biological and analytical variation. However, the subtle difference in the algorithm proposed by Tan *et al* is the recognition of the importance of changes in cardiac troponin concentration within the normal reference range. For example, consider a male patient with a high-sensitivity cardiac troponin I concentration of 10 ng/L at presentation and a concentration of 25 ng/L at 3 hours. Despite a >50% change in concentration, they would be ruled out by the 3-hour diagnostic pathway of the European Society of Cardiology if they had no ongoing chest pain and had a low GRACE score (<140). In the pathway proposed by Tan *et al*, this patient would be considered at high risk of myocardial infarction and would be recommended for further testing. This will reduce the likelihood of missed index diagnoses. While Tan *et al* recommend a relative change in cardiac troponin concentration, absolute changes in cardiac troponin concentration may also be informative at lower concentrations. An absolute change of >3 ng/L at 3 hours in those patients with troponin concentrations ≤99th centile at presentation identifies patients with increasing cardiac troponin concentrations who would benefit from further testing at 6 hours.^6^

The latest iteration of the European Society of Cardiology guidelines includes an alternative testing strategy using a presentation and 1-hour rule-out pathway with thresholds of cardiac troponin below the 99th centile.^4^ This strategy was derived and validated using a high-sensitivity cardiac troponin T assay, with secondary derivation of thresholds for cardiac troponin I, and has a high negative predictive value. As noted by Tan *et al*, laboratory turnaround times in some centres may prevent a clinician having a presentation troponin result within 1 hour, which they suggest may preclude use of this approach. One could argue that a serial sample may still be obtained at 1 hour although a delay in clinical decision-making would be necessary until both results were available. Current debate focuses on whether the thresholds for change in cardiac troponin I concentration as proposed in the 1-hour pathway of the European Society of Cardiology (≥2 ng/L) may be reliably distinguished from analytical variability, since as many as 50% of patients *without* acute coronary syndrome can have a change of 2 ng/L on serial testing.

While one of the major advantages of using a high-sensitivity assay is the earlier rule-out of myocardial infarction, accurate identification of patients *with* myocardial infarction is equally important as this may facilitate earlier treatment and transfer to an appropriate care setting. The Asia-Pacific consortium proposes a rule-in threshold for myocardial infarction of 10 times the upper limit of normal (260 ng/L for the Abbott ARCHITECT*_STAT_* high-sensitivity troponin I assay). This threshold was recommended by the Asia-Pacific consensus group, as the current European Society of Cardiology threshold of 52 ng/L only has a positive predictive value of 84%.^5^ The authors quote a specificity of 98% for their rule-in threshold of 260 ng/L, but the source of this is unclear. As a rule-in threshold will lead to treatment for myocardial infarction, the authors’ approach for maximising positive predictive value is justified although any patient with elevation >99th centile on presentation still requires serial testing to demonstrate a rise and fall: an integral component of the definition of myocardial infarction.

The authors recommend clinicians adopt gender-specific thresholds when using the Abbott ARCHITECT*_STAT_* high-sensitive cardiac troponin I assay, where concentrations in men are two-fold higher than those in women. Use of gender-specific thresholds identify women at higher risk of future myocardial infarction or cardiac death, and there is the potential for outcomes to be improved.^8^ Importantly, Tan *et al* highlight this difference in 99th centile concentration between men and women is consistent across different populations, despite the influence of age, renal function, ethnicity and specimen type.^3^

In settings where access to laboratory facilities is limited, Tan *et al* acknowledge there may be a role for contemporary point-of-care testing. It is imperative that patients with suspected acute coronary syndrome are not ‘ruled out’ on the basis of a normal or undetectable point-of-care result until repeat testing has been performed at 6 hours or the patient has undergone formal laboratory testing. We firmly agree with the authors' recommendation for an ongoing programme of education for those clinicians who are requesting and interpreting cardiac troponin tests. This is of greatest importance for new or rotating clinical staff who may be unfamiliar with the cardiac troponin assay in use at their new institution, as cardiac troponin decision thresholds and concentrations are not interchangeable between different assay types.

In summary, Tan *et al* should be commended for compiling a large volume of evidence into a clear and concise series of recommendations for the clinician who may be seeking to introduce high-sensitivity cardiac troponin testing into clinical practice. In this constantly evolving field, it is important that all clinicians remain abreast of the latest developments, with further changes in the recommended strategy for rule-out of acute coronary syndrome likely in the fourth iteration of the universal definition of myocardial infarction in 2018.
